# Supplementary material for: Evaluation of Structure Prediction and Molecular Docking Tools for Therapeutic Peptides in Clinical Use and Trials Targeting Coronary Artery Disease
Source: Int J Mol Sci. 2025 Jan 8;26(2):462. doi: 10.3390/ijms26020462 (PMC11765240; doi:10.3390/ijms26020462)
Supplement: Supplementary file 1 [file ijms-26-00462-s001.zip › Supplementary Data S1 - Evaluation of Structure Prediction Tools.pdf]

Supplementary Data 1
Protein Structure Analysis Results

Peptide	Z-Score	Overall Model Quality	Local Model Quality	Ramachandran Plot
**ANP**				
AlphaFold 3	-2.84	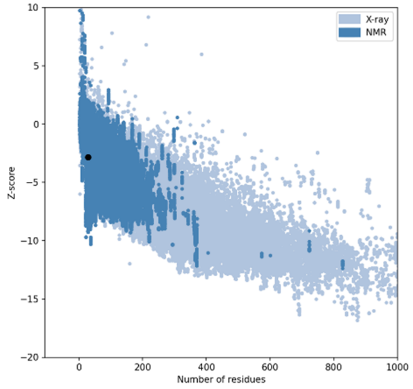	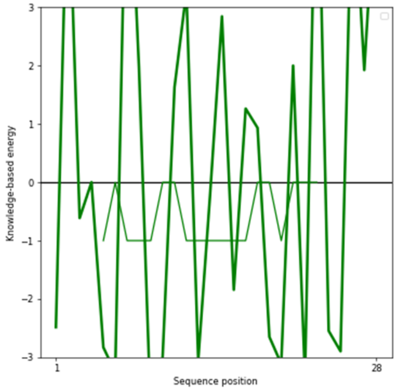	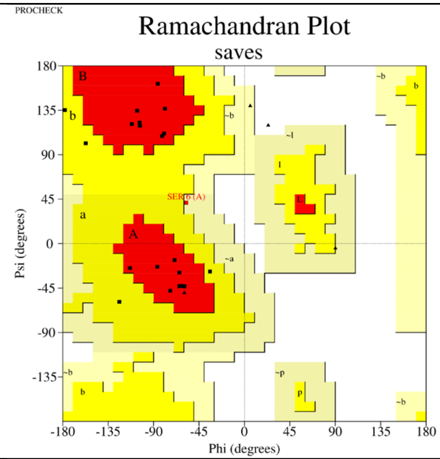
I-TASSER	-2.77	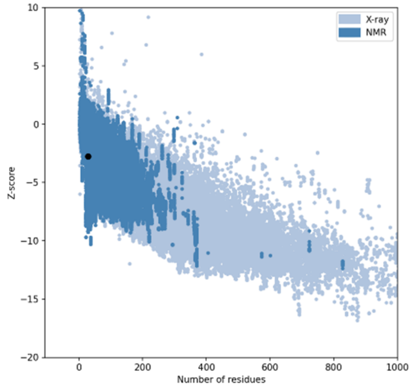	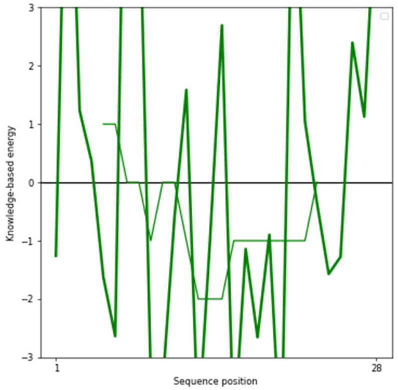	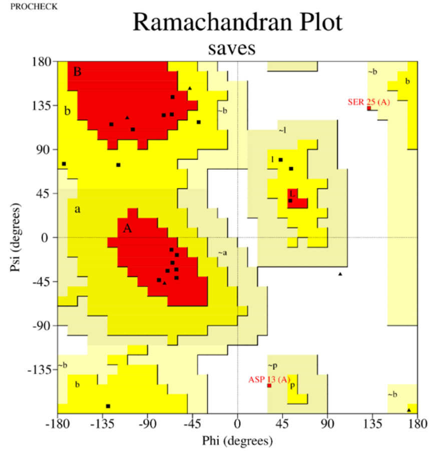

PEP-FOLD 4	-1.62	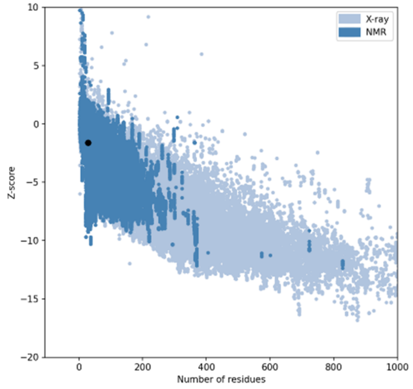	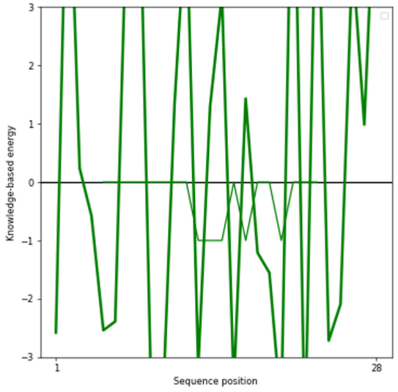	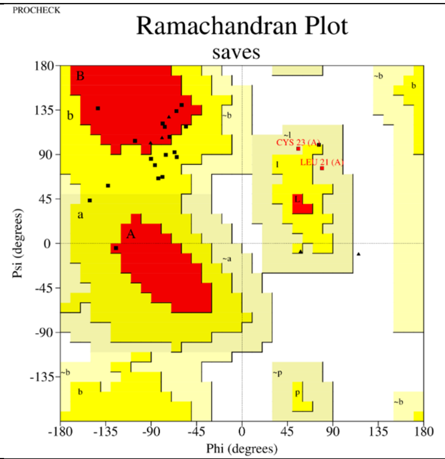
**Apelin**				
AlphaFold 3	-4.21	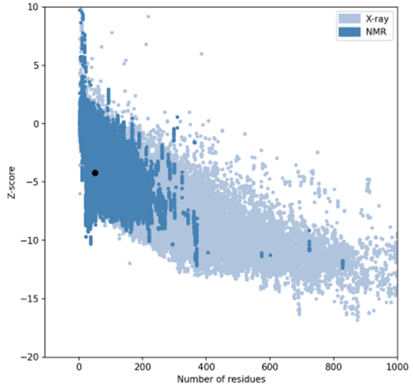	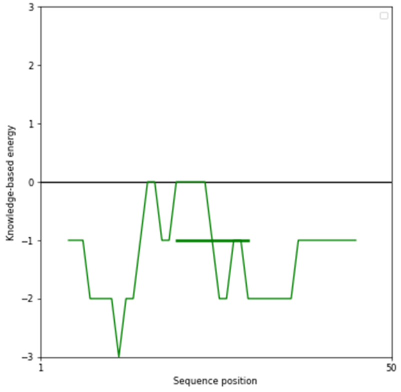	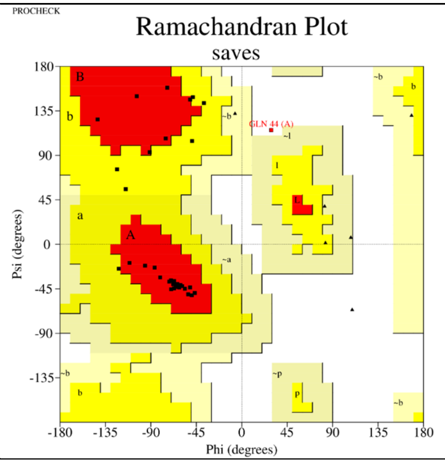

I-TASSER	-2.06	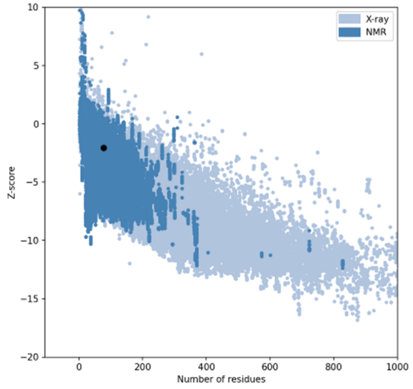	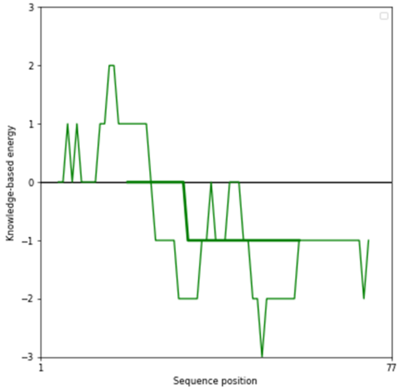	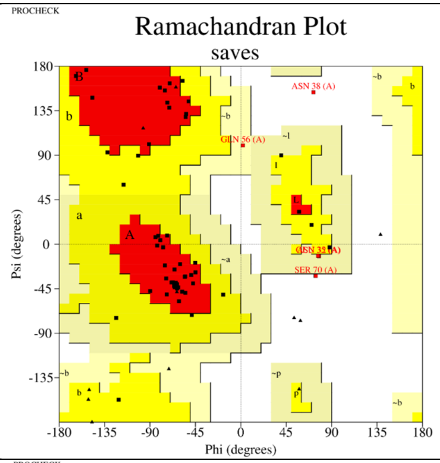
PEP-FOLD 4	-1.15	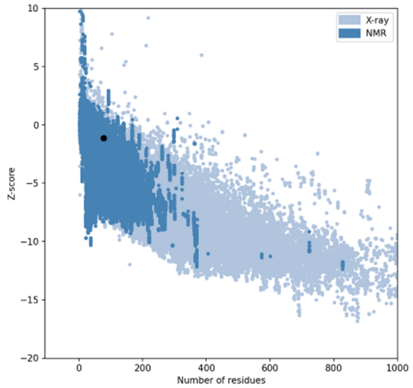	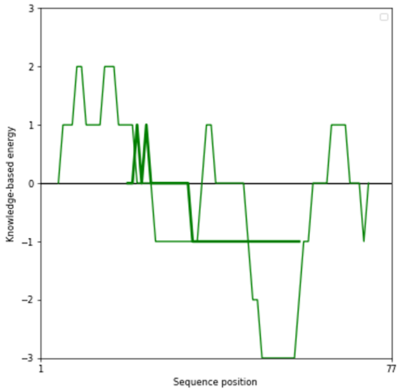	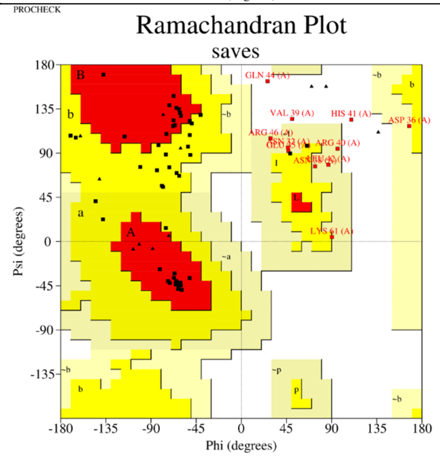

**Exenatide**				
AlphaFold 3	-3.68	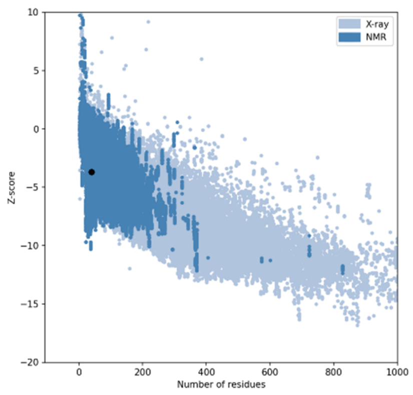	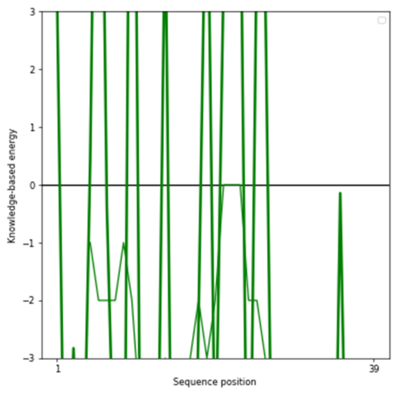	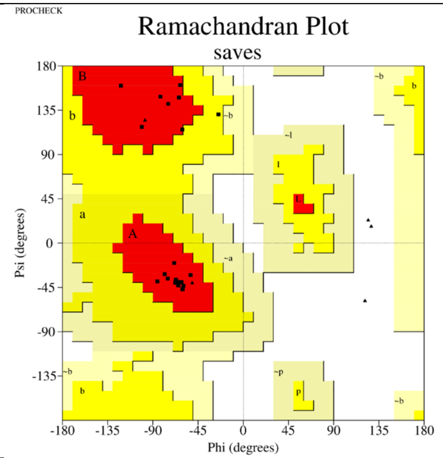
I-TASSER	-2.05	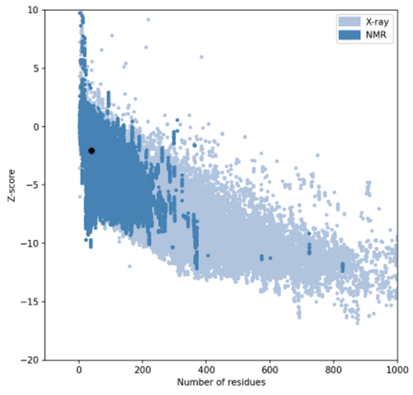	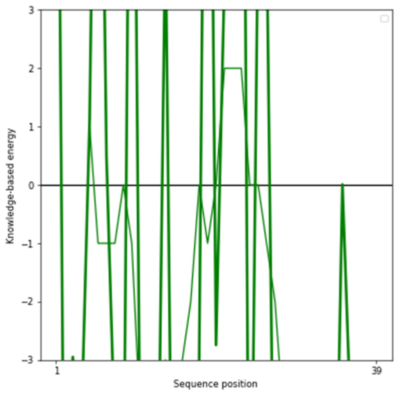	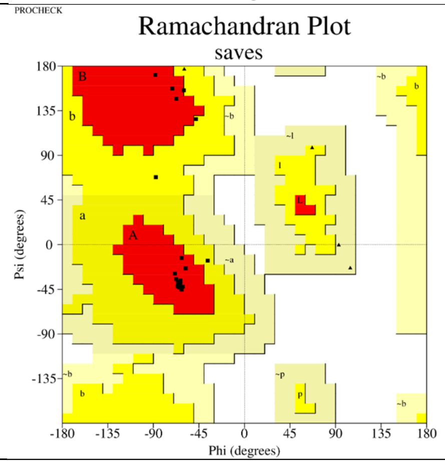

<table>
<tr><td>PEP-FOLD 4</td><td>-2.41</td><td>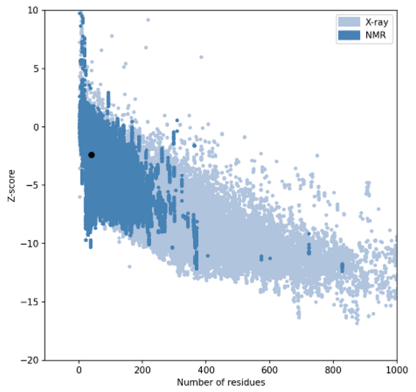
</td><td>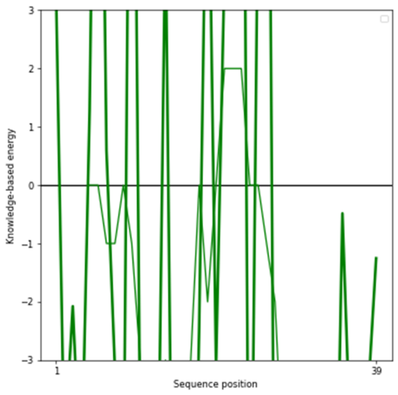
</td><td>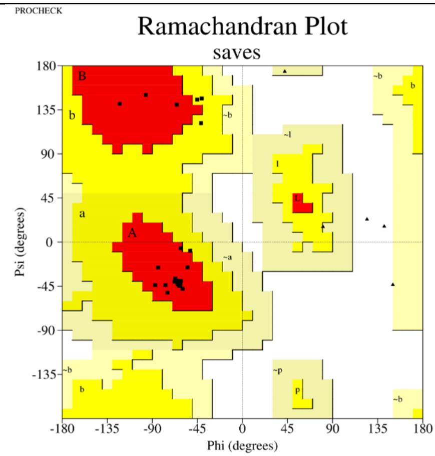
</td></tr>
<tr><td colspan="4">FX06</td><td></td></tr>
<tr><td>AlphaFold 3</td><td>-4.72</td><td>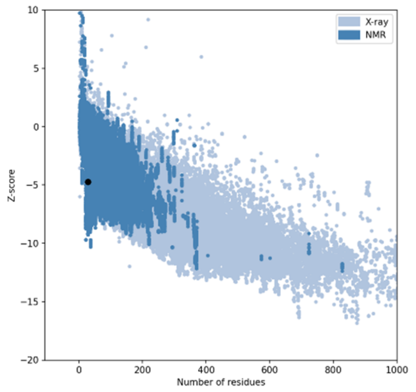
</td><td>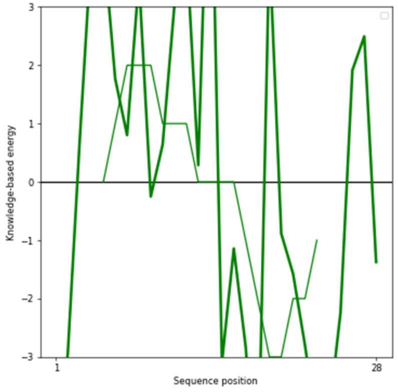
</td><td>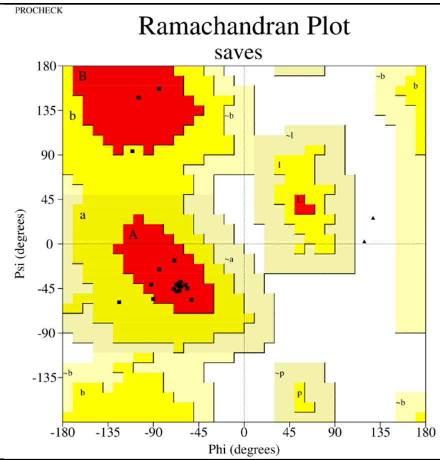
</td></tr>
</table>

<table>
<tr><td>I-TASSER</td><td>-4.46</td><td>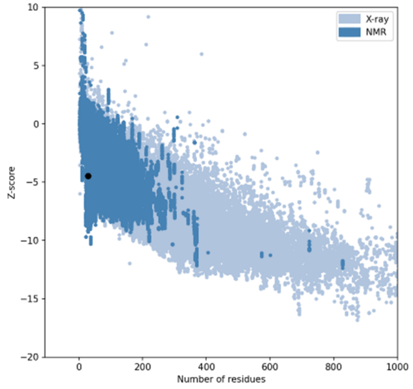
</td><td>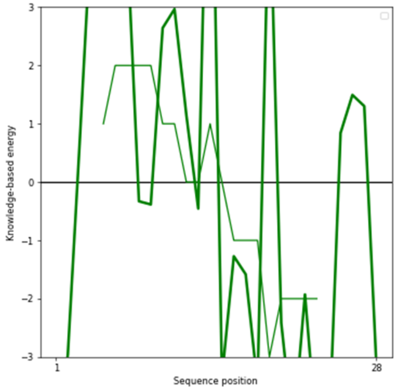
</td><td>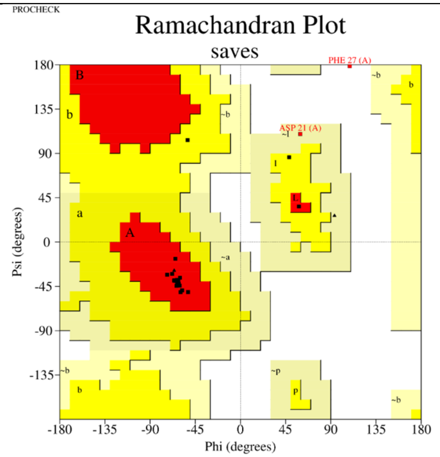
</td></tr>
<tr><td>PEP-FOLD 4</td><td>0.11</td><td>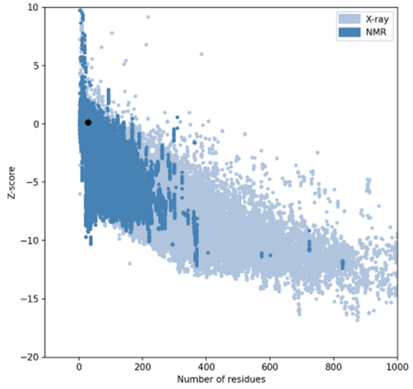
</td><td>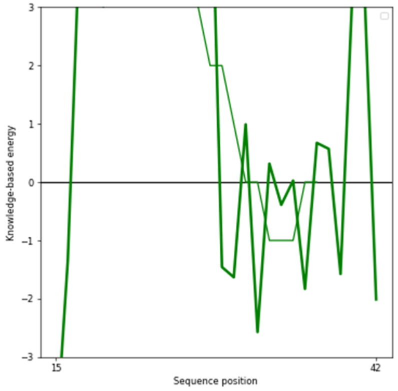
</td><td>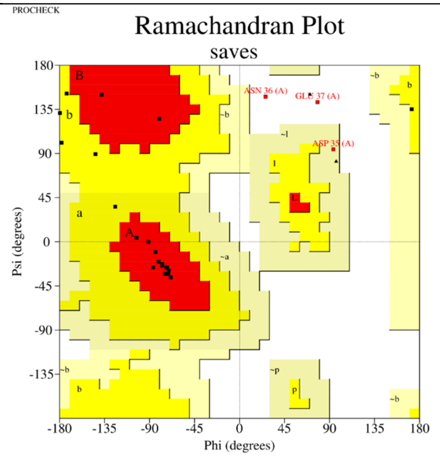
</td></tr>
</table>

<table>
<tr><td colspan="5">GLP-1</td></tr>
<tr><td>AlphaFold 3</td><td>-0.95</td><td>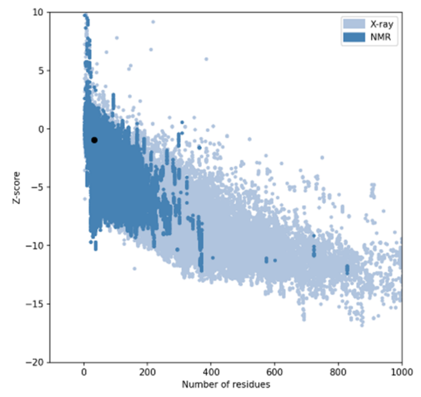</td><td>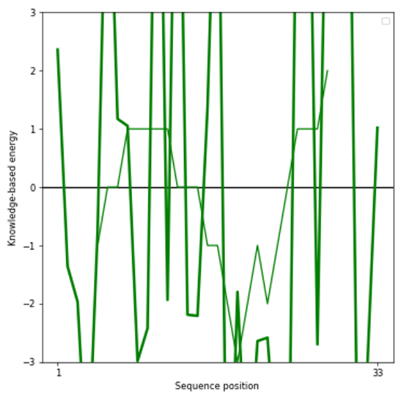</td><td>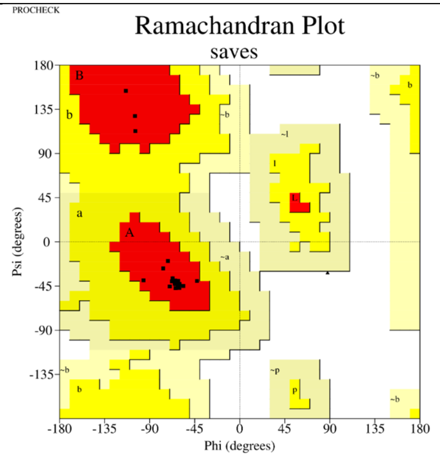</td></tr>
<tr><td>I-TASSER</td><td>-0.91</td><td>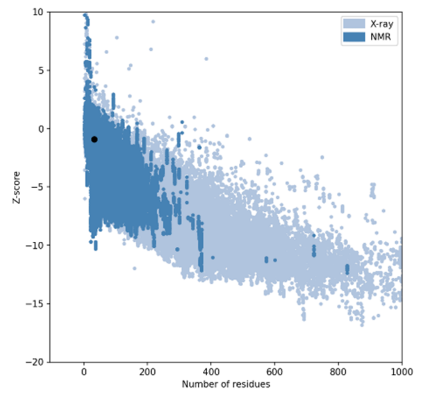</td><td>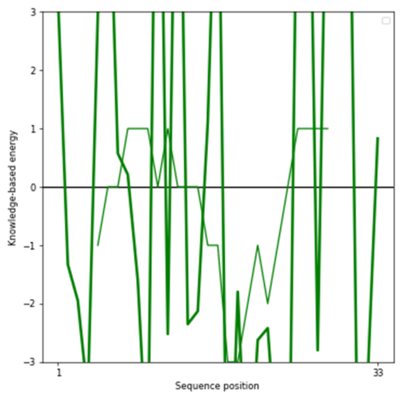</td><td>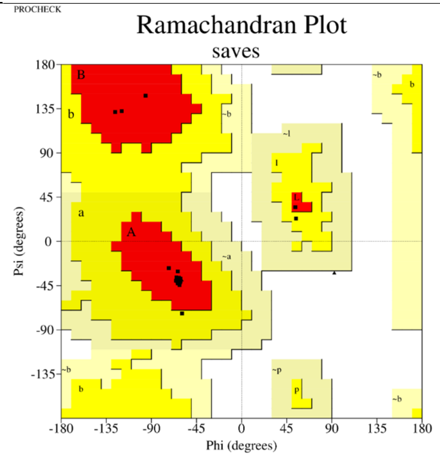</td></tr>
</table>

PEP-FOLD 4	-0.72	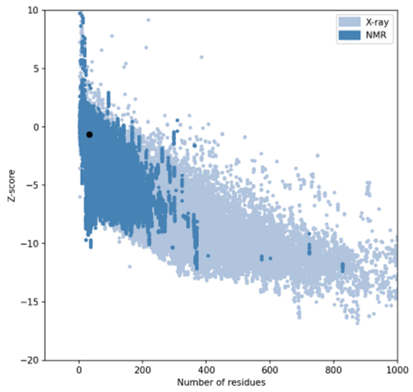	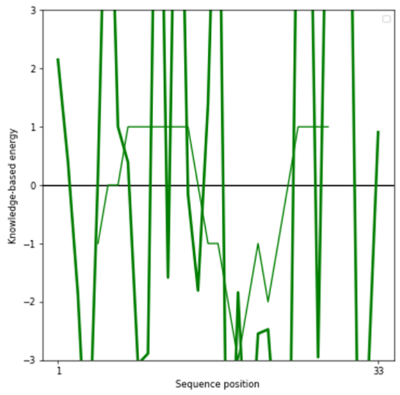	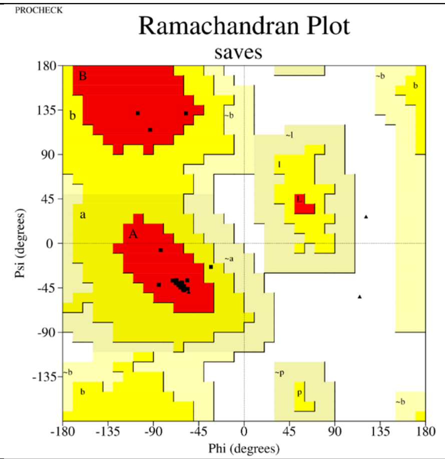
**Liraglutide**				
AlphaFold 3	-2.51	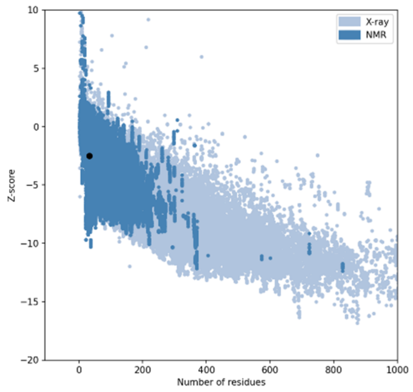	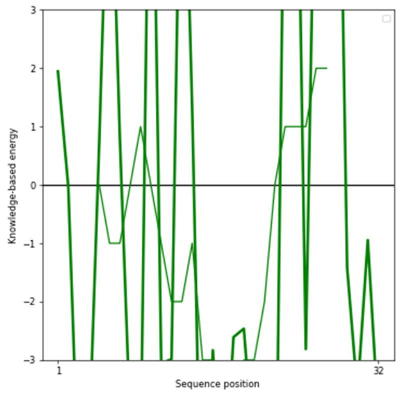	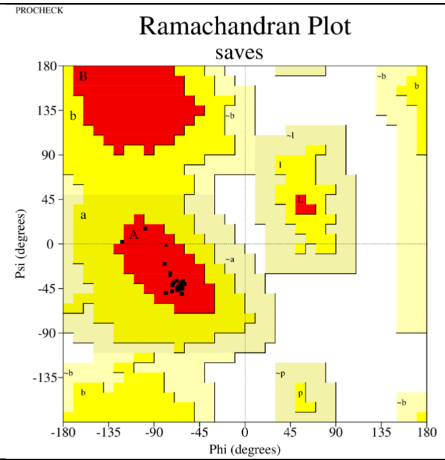

I-TASSER	-0.58	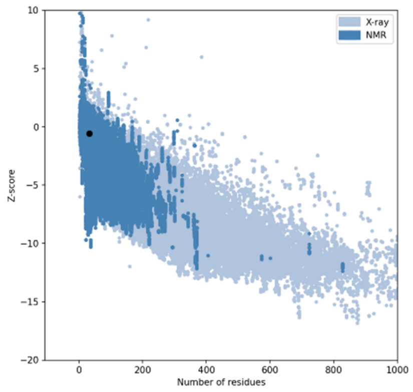	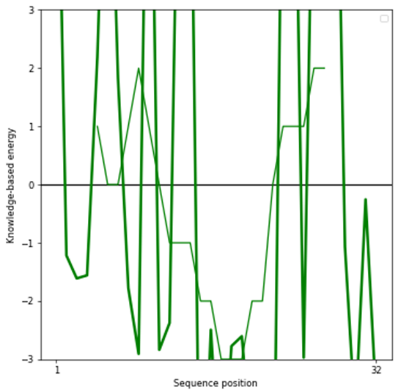	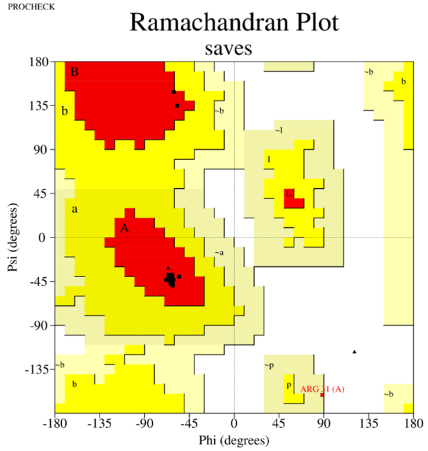
PEP-FOLD 4	-0.62	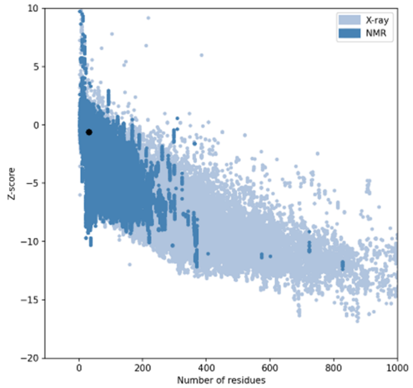	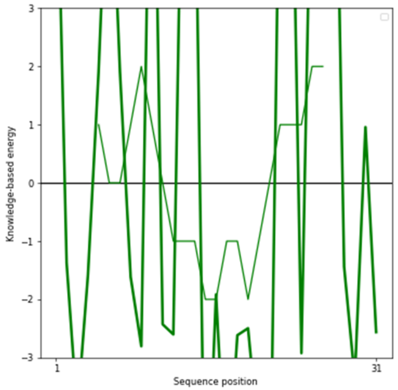	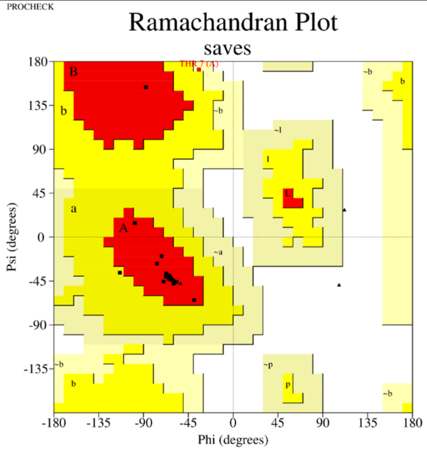

**Nesiritide**				
AlphaFold 3	-1.65	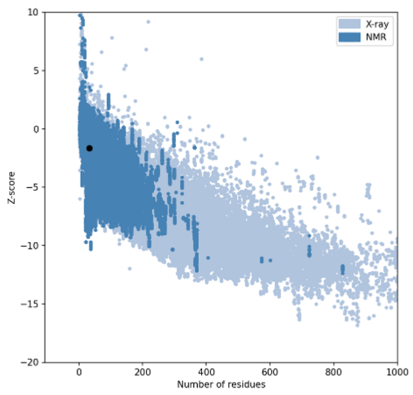	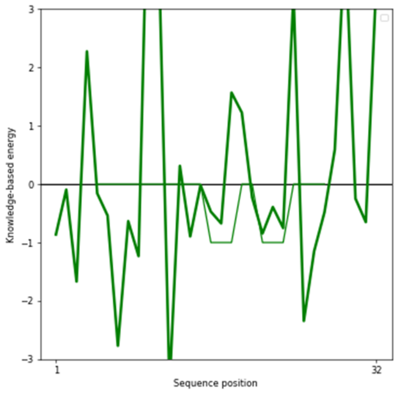	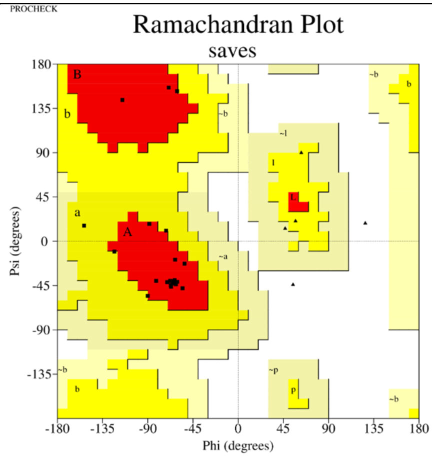
I-TASSER	-0.95	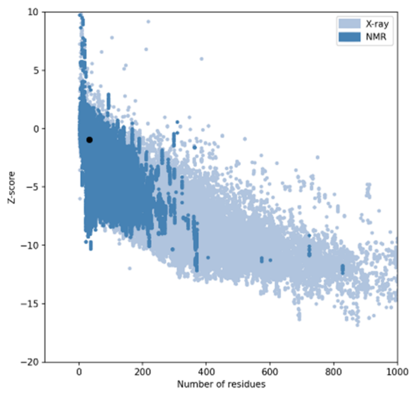	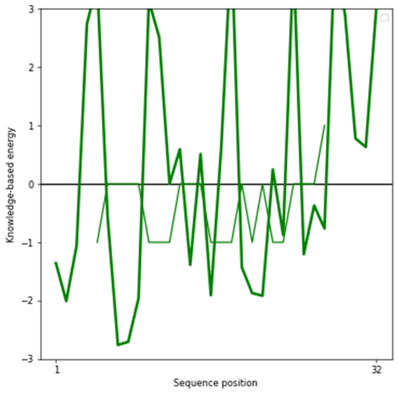	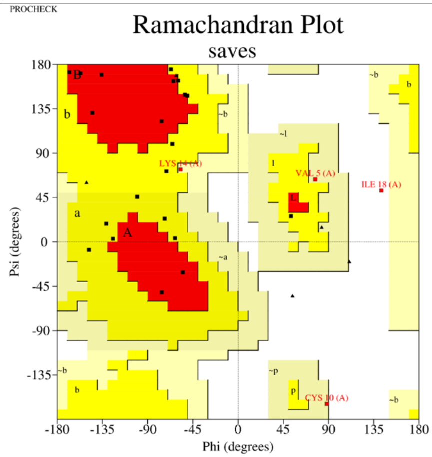

PEP-FOLD 4	-1.56	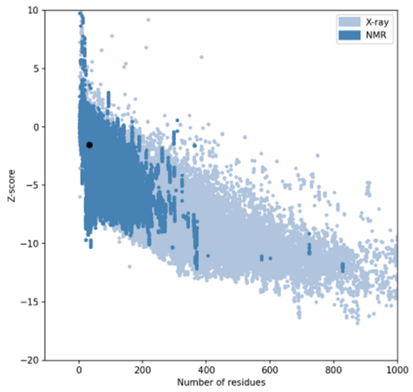	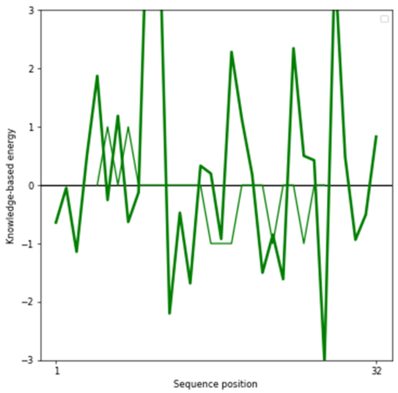	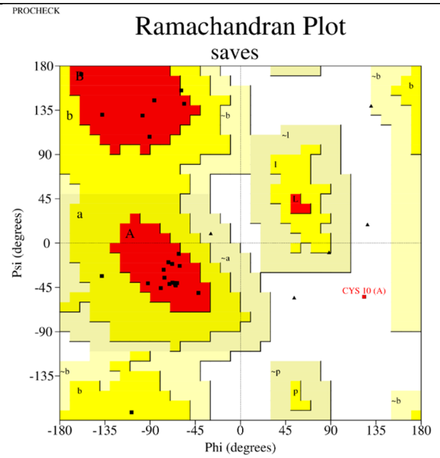
